# Revised estimates of ocean-atmosphere CO_2_ flux are consistent with ocean carbon inventory

**DOI:** 10.1038/s41467-020-18203-3

**Published:** 2020-09-04

**Authors:** Andrew J. Watson, Ute Schuster, Jamie D. Shutler, Thomas Holding, Ian G. C. Ashton, Peter Landschützer, David K. Woolf, Lonneke Goddijn-Murphy

**Affiliations:** 1 College of Life and Environmental Sciences, University of Exeter, Exeter, UK; 2 Max Planck Institute for Meteorology, Bunderstr. 53, 20146 Hamburg, Germany; 3 International Centre for Island Technology, Heriot-Watt University, Stromness, Orkney UK; 4 Environmental Research Institute, University of the Highlands and Islands, Ormlie Road, Thurso, UK

**Keywords:** Carbon cycle, Climate change, Marine chemistry

## Abstract

The ocean is a sink for ~25% of the atmospheric CO_2_ emitted by human activities, an amount in excess of 2 petagrams of carbon per year (PgC yr^−1^). Time-resolved estimates of global ocean-atmosphere CO_2_ flux provide an important constraint on the global carbon budget. However, previous estimates of this flux, derived from surface ocean CO_2_ concentrations, have not corrected the data for temperature gradients between the surface and sampling at a few meters depth, or for the effect of the cool ocean surface skin. Here we calculate a time history of ocean-atmosphere CO_2_ fluxes from 1992 to 2018, corrected for these effects. These increase the calculated net flux into the oceans by 0.8–0.9  PgC yr^−1^, at times doubling uncorrected values. We estimate uncertainties using multiple interpolation methods, finding convergent results for fluxes globally after 2000, or over the Northern Hemisphere throughout the period. Our corrections reconcile surface uptake with independent estimates of the increase in ocean CO_2_ inventory, and suggest most ocean models underestimate uptake.

## Abstract (web summary)

Ocean uptake of carbon dioxide impacts the climate, but flux estimates from surface measurements have not been corrected for temperature differences between surface and water sampling depth. Making that correction, the authors find previous estimates for ocean uptake have been substantially underestimated.

## Introduction

In recent years, an international effort has assembled a quality-controlled database of surface ocean carbon dioxide observations, the Surface Ocean Carbon Dioxide Atlas (SOCAT)^1–3^. SOCAT has enabled several recent studies evaluating air–sea CO_2_ flux from the observed partial pressure at the ocean surface^4–10^. In order to use the data to obtain accurate values of ocean-atmosphere CO_2_ fluxes, it is necessary to apply the gas exchange equation to the concentration difference of dissolved CO_2_ across the mass boundary layer (MBL) of the sea surface—the topmost ~100 µm within which molecular diffusion dominates vertical transport toward the interface (see Methods section). Calculation of the concentration at the base of this layer comes from the sea surface data. Previous studies using the SOCAT data have generated this concentration from the measurements specified at the temperature of the water inlet, at a depth usually several meters below the surface. However, there are typically temperature differences in the surface ocean layer^11^, which can materially affect the partial pressure, or fugacity of CO_2_ (fCO_2_). For this reason, a procedure was developed to recalculate the SOCAT data from the measurement temperature to the subskin temperature, a few millimeters below the surface^11–13^. This temperature is derived predominantly from satellite infrared observations and is available as an optimally interpolated gridded product^14^. Furthermore, the MBL is embedded within the ocean’s thermal skin, the uppermost ~1000 µm which is cooler than the underlying water because the ocean surface is a net emitter of heat, both via latent heat and longwave radiative fluxes, to the atmosphere^15^. The cooler skin temperature also affects the calculation of the CO_2_ flux since the CO_2_ concentration at the top of the MBL, at the air–water interface, is set by the product of the fCO_2_ in the atmosphere, and its solubility in the water there. The solubility is temperature dependent and increases at the lower temperature. It has long been known that this effect has a globally significant impact on calculated air–sea fluxes^16^, but most studies have ignored it. Recent work has however confirmed and clarified the theory^11^.

Here, we apply these corrections to a recent update of the SOCAT data, in combination with several different interpolation techniques. We derive a time history of corrected ocean-atmosphere fluxes and their associated uncertainties, for the period from 1992 to 2018, finding substantially increased net uptake of CO_2_ by the oceans. We then compare our results with a recently published analysis of the increase in ocean anthropogenic carbon dioxide calculated from global repeat hydrography programs^17^. In contrast to earlier surface flux estimates, our revision is consistent with this inventory increase. Comparison with the inventory suggests that the pre-industrial flux of CO_2_ from the open ocean to the atmosphere was ~0.5 PgC yr^−1^ and that it exhaled mostly from the Southern Hemisphere. The close agreement between two independent observationally based syntheses, one based on surface data and the other on interior measurements, suggests that most ocean carbon models are underestimating the net sink for atmospheric CO_2_ over recent decades.

## Results and discussion

### Effect of temperature corrections

**Fig. 1:**
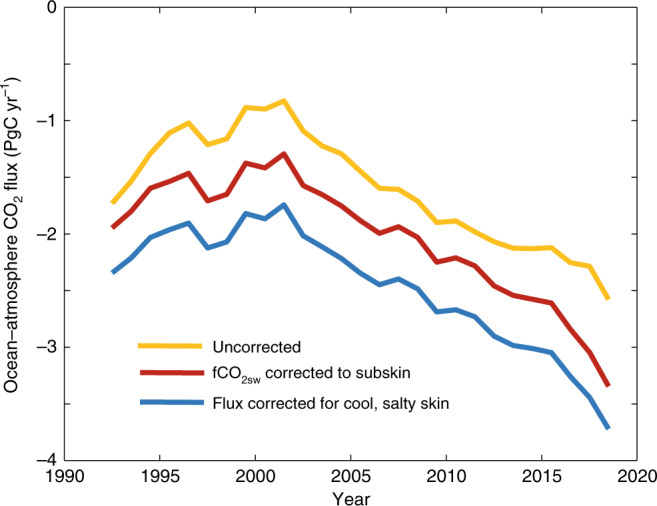
Effect of near-surface temperature corrections. Global air–sea flux calculated by interpolating SOCAT gridded data using a neural network technique^8^, followed by the gas exchange equation applied to the ocean mass boundary layer. The net flux into the ocean is shown as negative, following convention. The uncorrected curve uses the SOCAT fCO_2_ at inlet temperature as usually done. Correction of the data to a satellite-derived subskin temperature is shown, and the additional change in flux due to a thermal skin assumed to be cooler and saltier than the subskin by 0.17 K^15^ and 0.1 salinity units^11^. Excludes the Arctic and some regional seas—ocean regions included are shown in Supplementary Fig. 2.

Figure 1 illustrates the effect of the two adjustments described above on a calculation of annual global ocean-atmosphere fluxes for this period, with calculations starting from the SOCAT v2019 database. To interpolate the SOCAT surface water fCO_2_ data in space and time we adopt as our standard method the two-step neural network approach described by Landschützer et al.^8,18^, (see also description below and Methods section). The interpolation was applied to the SOCAT data without modification, after adjusting the data to a subskin temperature and regridding (as described in refs. ^12,19^, see also Methods section) then additionally after repeating the flux calculation assuming a Δ*T* across the cool skin of 0.17 K^15^ salinity increase of 0.1 unit^11^ and the conservative “rapid transport” scheme of Woolf et al.^11^ (see Methods section). Each adjustment increases the calculated flux by ~0.4 PgC yr^−1^ when integrated over the global ocean. For the period ~2000, this approximately doubles the calculated flux into the ocean. Over the 27 years 1992–2018 inclusive, the cumulative uptake is increased from 43 to 67 PgC.

### Uncertainty estimates

Ocean-atmosphere fluxes calculated using the gas exchange equation are subject to two broad sources of uncertainty: (1) specification of the gas transfer velocity, which depends on the thickness of the MBL and is usually parameterized as a function of wind speed, and (2) specification of the CO_2_ concentration difference across the MBL. The recent study by Woolf et al.^20^ contains a detailed treatment of the uncertainties due to the gas transfer, concluding that a realistic estimate (approximately, a 90% confidence interval) is ±10% when applying this to global data.

The second source of uncertainty, due to the concentration difference, is dominated by that introduced by the interpolation in time and location of surface ocean CO_2_. This is relatively well constrained in the more densely observed regions such as the North and Equatorial Atlantic and North and Tropical Pacific. However, in more remote regions such as the Southern, South Pacific, and Indian Oceans, the observational coverage is patchier in space and time and often seasonally biased, with few winter measurements (see Supplementary Fig. 3). New sensors and designs of autonomous floats, as now being deployed in the Southern Ocean^21^, show promise to solve the problem of adequately observing surface CO_2_ in remote regions^22^, but for the gap-prone historical data, the interpolation method used can have a substantial influence on the results in these data-poor regions.

To evaluate the uncertainty in flux estimates introduced by the gap-filling procedure, we used three methods for interpolating in space and time, each applied to the global data divided according to three different spatial clustering schemes, for a total of nine mappings. The interpolation methods were as follows: (1) a time series (TS) of fCO_2sw_ data, constructed by a least squares fit to all monthly averaged fCO_2_ values within the defined region. The model fitted was a seasonal cycle with three harmonics superimposed on a linear trend; (2) simple multilinear regression (MLR) of the fCO_2_ data on latitude, longitude, and four variables for which continuous comprehensive mappings are available, these being sea-surface temperature (SST), salinity (SSS), mixed layer depth (MLD), and atmospheric CO_2_ mixing ratio (XCO_2_); (3) the feed-forward neural network method of Landschützer et al.^8,18^ (FFN), which also seeks a regression on these four variables. The spatial clustering schemes applied to each of the techniques (shown in Supplementary Fig. 1) were as follows: (a) division into 14 regions along latitude–longitude lines; (b) division into the 17 biogeochemical divisions suggested by Fay and Mckinley^23^, and (c) division into 16 biomes using a self-organizing map technique employed by Landschützer et al.^8^.

Where the data are adequately distributed over space and time, the use of multiple mapping techniques and different clustering schemes to estimate uncertainty gives similar results to formal geostatistical techniques, such as kriging^7,20^. However, in regions of very sparse and uneven coverage, statistically based techniques can underestimate uncertainties because of the assumption that the available data are representative of the true data population over a region, which may not be the case if whole regions or seasons are poorly sampled. In this instance, different mapping techniques can give substantially different results. Altering the clustering of the data by changing the shape of the geographical divisions can also have a major effect, because unsampled areas are assumed to have the same statistical properties as the sampled regions with which they are grouped.

**Fig. 2:**
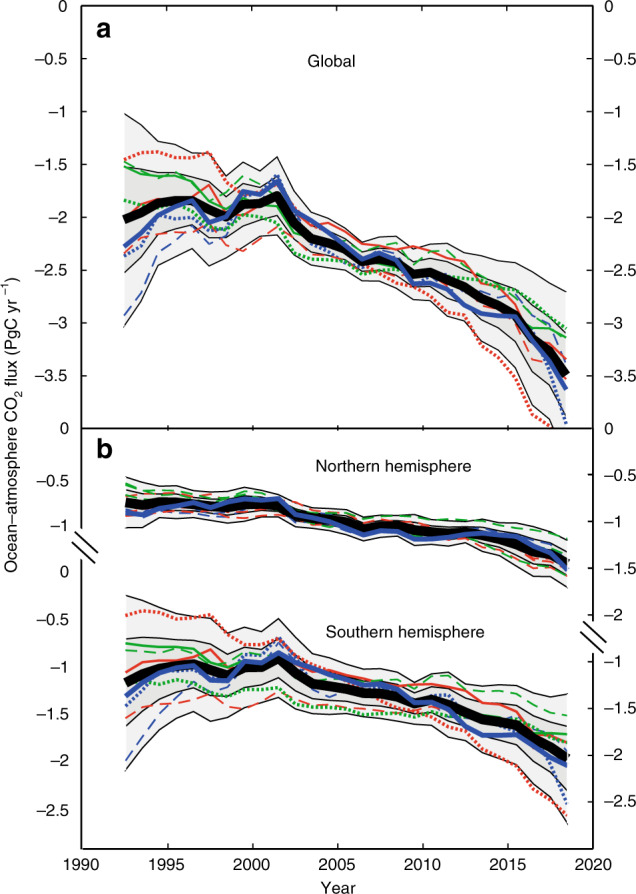
Global ocean-atmosphere CO_2_ fluxes 1992–2018. Fluxes are integrated **a** globally, and **b** for northern and southern hemispheres, calculated using a standard gas exchange formulation (see Methods section) with the nine interpolation schemes for fCO_2_ described in the text shown as colored lines: TS red, MLR green, FFN blue. The line styles indicate the spatial clustering schemes used (illustrated in Supplementary Fig. 1): solid, Landschützer SOM; dashed, latitudinal regions; dotted, Fay and Mckinley biomes. The standard method, SOM-FFN as described in Landschützer et al.^8^, is shown as a thicker blue line. Shading indicates one- and two-standard deviations of the nine methods around the mean (thick black line).

For each combined mapping-and-clustering technique, Table 1 shows the spread and mean of the residuals (the global set of predicted values minus observed values). The neural network FFN mapping method provides a much smaller spread of residuals, giving better agreement with data at a given location and time than do the other methods. This is to be expected given its much greater flexibility, with typically several hundred parameters being adjusted to provide a non-linear fit to each cluster, compared to only 8 and 11 fitted parameters for respectively the TS and MLR methods. Figure 2 shows estimates of global and hemispheric ocean-atmosphere CO_2_ flux over the period 1992–2018 by the nine interpolations (using a single parameterization of the gas transfer velocity). Despite the difference in the quality of the fits to the individual data as evidenced by Table 1, convergent results are obtained by all the calculations for the Northern Hemisphere over the whole period, and there is a good agreement in the Southern Hemisphere for much of the period after 2000. The average of all the methods is shown, with one and two standard deviations of the nine separate estimates. A few regions are excluded (see Supplementary Fig. 2) to ensure compatibility in the comparison between methods, but these affect the results by <0.05 PgC yr^−1^_._

**Table 1:** Statistics of the residuals of the predictions to data.

Method	Areal division	Interpolation method	Half-width of Gaussian *σ* (μatm)	Bias *b* (μatm)
1	Latitudinal regions	TS	23.6	1.37
2	Fay and McKinley biomes	TS	21.0	1.69
3	Landschützer SOM	TS	15.7	0.82
4	Latitudinal regions	MLR	21.8	1.86
5	Fay and McKinley biomes	MLR	18.0	0.72
6	Landschützer SOM	MLR	14.8	0.95
7	Latitudinal regions	FFN	11.3	0.37
8	Fay and McKinley biomes	FFN	10.3	0.27
9	Landschützer SOM	FFN	12.2	0.60

Residuals for the nine combinations (three interpolation methods each applied to three areal divisions or clustering, as shown in Supplementary Fig. 1). Gaussian curves$G\left( x \right) = A{\rm{exp}}\left\{ {\frac{{\left( {x {\,} - {\,} b} \right)^2}}{{2\sigma ^2}}} \right\}$ were fitted to the histograms of residuals *x*, where *A*, *b,* and *σ* are parameters determined by non-linear least squares. The bias *b* and the width *σ* for all the fits are given.

The wider uncertainties indicated pre-2000 arise from the divergence of fits in the Southern Hemisphere. The majority of studies using the historical surface CO_2_ data find that the Southern Ocean sink was static or weakening during the 1990s and strengthened considerably after 2000^24,25^. The simpler, linearly constrained interpolation methods show something of this change, but it is less pronounced than in the more flexible FFN calculations. However, we retain the wider spread pre-2000 as a realistic estimate of uncertainty then, given the paucity of the data and its uneven, and decadally changing, spatial distribution in the Southern Ocean and South Pacific (see Supplementary Fig. 3, which shows the distribution of the data in the Southern Hemisphere).

### New CO_2_ surface fluxes compared to interior observations

**Fig. 3:**
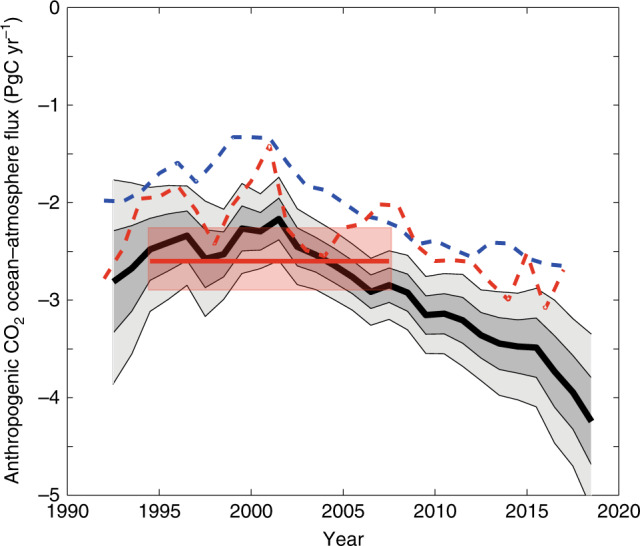
Observation-based estimates of anthropogenic CO_2_ uptake. The black line is our standard case global ocean-atmosphere flux increased by −0.57 PgC Cyr^−1^ to account for pre-industrial and Arctic fluxes as described in the text. The shading gives one and two standard deviations of estimates around this value, including the uncertainty in gas transfer rates as assessed by Woolf et al.^20^. Red horizontal line and uncertainty is a recent estimate of the global inventory increase of anthropogenic carbon in the ocean between 1994 and 2007^17^. Dashed lines: two previous estimates of global uptake based on surface data: blue dashed line from Landschützer et al.^8^, red dashed line from Rödenbeck et al.^10^, both as quoted in Le Quéré et al.^31^. Both are increased by the pre-industrial flux correction and Landschützer et al.^8^ also increased by Arctic correction.

We now compare our estimates for global CO_2_ flux into the ocean, with a recent independent synthesis of observations estimating the increase in oceanic anthropogenic carbon from interior repeat hydrography measurements, over the period 1994–2007^17^. This comparison requires accounting for pre-industrial ocean-atmosphere fluxes: the ocean was pre-industrially a source of CO_2_ to the atmosphere, with a net dissolved river flux usually estimated as 0.45–0.6 PgC yr^−1^ flowing down rivers to the ocean, from ocean to atmosphere and from the atmosphere to the land surface^26,27^. We also have to add a flux for the Arctic Ocean, not included in our study but estimated at 0.12 PgC yr^−1^ ^28^. In Fig. 3, we show our standard case estimate of the anthropogenic sink, with the ocean-atmosphere flux increased by 0.57 PgC yr^−1^, (0.12 PgC yr^−1^ Arctic plus a pre-industrial flux of 0.45 PgC yr^−1^), and with the uncertainty bands now widened to include the Woolf et al. estimate for gas transfer velocity uncertainty^20^. This is compared to the recent estimate for the accumulation of anthropogenic carbon from interior ocean observations, over the period 1994–2007^17^. Two previously published estimates of the sink calculated from the surface data are also shown for comparison^8,10^. In contrast to these earlier estimates, our revised surface flux is consistent with the interior anthropogenic accumulation and most previous estimates of the pre-industrial ocean-to-atmosphere source.

In Table 2, we show uptake integrated over the 13 years from mid-1994 to mid-2007 in the northern and southern Pacific, Indian and Atlantic basins, and compare these with the inventory increases as given by Gruber et al.^17^. The inventory increase in each basin will not equal the flux through the surface of that basin, both because of the pre-industrial flux correction described above, and because subsurface ocean transport redistributes the CO_2_ away from the uptake regions. The comparison is revealing, however, because we should not expect a very large change in inter-hemispheric CO_2_ exchange in the ocean during this time. We expect some correspondence between these figures, therefore, at least at the hemispheric level. The global flux through the ocean surface is less than the inventory change by ~7 PgC over this period, an amount consistent with the expected pre-industrial ocean source. However, the Northern Hemisphere uptake, which is comparatively well constrained by the surface data, quite closely matches the inventory increase in the Northern Hemisphere. The majority of the difference between surface uptake and inventory increase is in the Southern Hemisphere, suggesting that excess river carbon that the natural cycle puts into the open ocean was pre-industrially compensated by net outgassing almost entirely in that hemisphere, and that its magnitude is ~0.5 PgC yr^−1^. A recent proposed upward revision of this flux to 0.78 Pg Cyr^−1^ ^29^ was motivated in part by the clear mismatch between anthropogenic carbon uptake and the earlier, lower estimates of surface uptake, but our analysis is more consistent with the lower values of previous studies, which come from ocean inverse models^26^ and inventories of global dissolved riverine carbon^27,30^. We note also that the uncertainty on the South Pacific flux (including the Pacific sector of the Southern Ocean) is particularly large, reflecting the paucity of data there (Supplementary Fig. 3). However, over the entire Pacific basin, and globally, uncertainties are smaller, because there is inter-basin compensation with some mapping estimates that give high values in the southern Pacific giving lower values in the tropical and northern regions.

**Table 2:** Estimates of ocean CO_2_ uptake compared to interior inventory of anthropogenic carbon.

	Atlantic	Pacific	Indian	Other regions	Global
Cumulative CO_2_ uptake through surface (−ve is into ocean) July 1994 to June 2007 (PgC, ±2*σ*)					
North	−5.68 ± 0.97	−6.60 ± 0.90	+1.16 ± 0.43	−1.56 ± 0.8	−12.7 ± 1.6
South	−3.22 ± 0.91	−3.43 ± 4.6	−7.41 ± 0.96	–	−14.1 ± 4.6
Total	−8.91 ± 1.50	−10.04 ± 4.3	−6.25 ± 1.20	–	−26.8 ± 3.4
Gruber et al.^17^ estimates of inventory increase 1994–2007 (PgC)					
North	6.0 ± 0.4	5.2 ± 0.6	0.8 ± 0.4	1.5 ± 0.6	13.5 ± 1.0
South	5.9 ± 1.2	8.0 ± 1.2	6.3 ± 3.4	–	20.1 ± 3.8
Total	11.9 ± 1.3	13.2 ± 1.3	7.1 ± 3.4	–	33.7 ± 4.0

The agreement between the observational estimates of CO_2_ uptake by the oceans provides an important constraint on calculations of the global carbon budget and its rate of change. Supplementary Table 1 gives more detail on global fluxes calculated over decadal periods compared to those of earlier estimates, both of the net contemporary ocean-atmosphere flux and the ocean uptake of anthropogenic carbon. As indicated in Fig. 3, our best estimate of 2.5 ± 0.4 Pg yr^−1^ for anthropogenic carbon uptake during 1994–2007 agrees closely with that of Gruber et al. and has a similar uncertainty. This sink is stronger than most recent estimates and is ~0.5 PgCyr^−1^ larger than the central estimate of the Global Carbon Project^31^ for that period for example. That estimate is the average of a number of models, which however span a wide range, with a 2-*σ* uncertainty of ±0.6 PgC yr^−1^ for that period. The discrepancy between our value and that of the Global Carbon Project increases with time and approaches 1 PgC yr^−1^ after 2010.

We conclude that, when correctly applied, two data-led independent estimates for the ocean sink for CO_2_, based respectively on observations of the surface flux and the interior inventory of CO_2_, agree within relatively well-constrained uncertainties. The sink so determined is larger than most ocean carbon models predict, and suggests that some revision of the global carbon budget is required. Due weight should be given to the constraints that ocean interior and surface observations impose when calculating global carbon budgets, and near-surface temperature deviations need to be taken into account when using surface observations to calculate fluxes. Continued systematic observation of the surface and interior ocean carbon system remains essential to tracking how the global carbon cycle is changing in response to human activities.

## Methods

### Adjustments to surface CO_2_ data

The individual voyage surface CO_2_ data (v2019) were downloaded from the SOCAT website (www.socat.info^1,2^). We used only the data from 1992 onward, since the number of observations begins to increase substantially at around that time^2^. We used the surface fCO_2_ product, which is closely equivalent to the partial pressure of CO_2_ in equilibrium with seawater but is the more correct variable for concentration and flux calculations^32^.

The SOCAT database records fCO_2_ of surface seawater measured in an equilibrator, temperature-corrected to an inlet temperature using an empirical equation^33^. As described in Goddijn-Murphy et al.^12^, the SOCAT inlet temperature is not the same as the temperature measured at the base of the MBL, the topmost ~100 µm of the ocean, which is where the water-side concentration must be specified for the purposes of air–sea flux calculations^11^. We used the “FluxEngine” programs (http://www.oceanflux-ghg.org/Products/FluxEngine^13,34^) to correct the SOCAT data to a standard subskin temperature derived from satellite optimally interpolated sea-surface temperature data, implementing the method described by Goddijn-Murphy et al.^12^, and assuming an isochemical temperature correction. A new “cruise-weighted” gridded product following the SOCAT methodology was then created using the corrected cruise data^3^. This produces a 1° longitude and latitude by one-monthly time resolution data set^3^. This gridded product is publically available^19^.

### Interpolation techniques for the global fCO_2_

These have typically involved two steps. First the full data set is divided into clusters (using location, time, or some other criteria), then an interpolation procedure is applied to each of these clusters. We used three different schemes for the initial division, and three methods of interpolating the fCO_2_ data within those clusters. The divisions used are described in the main text and illustrated in Supplementary Fig. 1. Permanently ice-covered regions, the Arctic ocean, coastal regions, and other areas unclassified in the Fay and Mckinley description of biomes^23^ were excluded from all techniques when comparing the output of different methods, (e.g., Fig. 2 and interpolation uncertainty estimates) to ensure a like-for-like comparison. Supplementary Figure 2 shows the regions excluded and included when making the comparisons and when calculating a best estimate using our standard method, e.g., for Fig. 3 and Table 2.

The three classes of method used to interpolate the fCO_2_ data within these clusters were as follows:

1. TS curves fitted to the mean of all data in the region binned into monthly time steps, using least squares. The curves were the sums of a linear trend and three sinusoidal cycles having frequencies of one, two, and three cycles per year. Each fit therefore had eight variable parameters: amplitude and phase for each sine curve, and slope and intercept for the inter-annual trend.
2. MLR of surface fCO_2_ with the co-located SST, SSS, MLD, and XCO_2_, latitude and longitude. The first four of these were each decomposed into two components: a climatology calculated as the averages over each month of the year for the period 1992–2018, and an anomaly from that climatology. In total, there were 10 variables therefore, which with a constant, yielded 11 parameters to be fitted by least squares. Table S1 in the supplementary information details the sources of these “driver” variables that we used. Surface chlorophyll derived from satellite ocean color is frequently used as a predictor variable for fCO_2_ interpolations^25^, but we preferred not to use this as it is not available before 1997, or in polar regions in the winter.
3. The FFN network method as described by Landschützer et al.^8^, implemented with the MATLAB neural net toolbox. The independent variables were again SST, SSS, MLD, and XCO_2_, decomposed as for the MLR into a seasonal climatology and anomalies from that climatology.

### Calculation of air–sea fluxes

The gas exchange equation was used to calculate *F*_CO2_, the sea-to-air flux of CO_2_, (positive from sea to air):

$$F_{{\mathrm{CO}}_{2}} = k.\left( {C_{{\mathrm{sw}}} - C^\prime } \right),$$

where *k* is the appropriate gas transfer velocity, *C*_sw_ is the concentration of dissolved CO_2_ at the base of the MBL and *C*′ is the concentration at the interface with the atmosphere. *C*′ was calculated as *α*_skin._*fCO*_2atm_, the product of the solubility of CO_2_ at the surface skin temperature and salinity (*α*_skin_) and *fCO*_2atm_, the fugacity of atmospheric CO_2_. The gradient from the base to the top of the thermal skin was assumed to be 0.1 salinity units^11^ and −0.17 K^15^. *C*_sw_ was calculated as *α*_subskin._*fCO*_*2*subskin_, the product of the seawater *fCO*_*2*_ corrected to the subskin temperature derived as described above, and the solubility at the subskin temperature and salinity. This treatment follows that by Woolf et al.^11^, implementing their “rapid transport” approximation for carbonate equilibration in the surface layers. In this approximation, the transport from the interior across the thermal boundary to the MBL is assumed to occur more rapidly than the time scale for reaction of CO_2_ with H_2_O molecules, so that the dissolved CO_2_ concentration does not change. This is a conservative assumption, in the sense that it gives a smaller adjustment due to skin effects than if change in the hydration state is assumed.

The gas transfer velocity was parameterized as a function of the wind speed at 10 m (we used the relation of Nightingale et al.^35^ based on a compilation of dual tracer experiments, which is one of several evaluated by Woolf et al.^20^ that give similar results to more recent parameterizations using the global ^14^C budget^36,37^). The wind used was the CCMP product at 0.25^o^ and 6-h resolution^38^, with gas exchange rates subsequently averaged monthly over 1 × 1 degree tiles. Atmospheric fugacity was calculated from XCO_2_, the atmospheric mixing ratio of CO_2_, using the method outlined for the CO_2_—air mixture by Weiss^32^, and assuming air at 100% humidity at the sea surface^39^. The sources of data for winds, surface temperature, salinity, atmospheric pressure, ice cover, and XCO_2_ used are given in Supplementary Table 1. Ice cover was assumed to suppress air–sea exchange entirely, so that the calculated flux was reduced by a factor (1 − *i*) where *i* was the fractional ice cover.

## Supplementary information

Supplementary Information

Peer Review File

Supplementary Data

## Data Availability

The gridded data set of sea surface fCO_2_ described in the Methods section above, based on SOCAT v2019 and adjusted to satellite-derived subskin surface temperature, is available at 10.1594/PANGAEA.905316. Ocean-atmosphere fluxes interpolated to monthly and 1 × 1 degree spatial resolution, and used to construct figures and tables in this publication, are available on request from the corresponding author. The mean annual fluxes used to draw the line graphs of Figs. 1–3 are included in the Supplementary Data file linked to this publication.
