# Supplementary material for: Revised estimates of ocean-atmosphere CO2 flux are consistent with ocean carbon inventory
Source: Nat Commun. 2020 Sep 4;11:4422. doi: 10.1038/s41467-020-18203-3 (PMC7474059; doi:10.1038/s41467-020-18203-3)
Supplement: Supplementary file 1 — Supplementary Information [file 41467_2020_18203_MOESM1_ESM.pdf]

Supplementary Information for

"Revised estimates of ocean-atmosphere CO_2 flux are consistent with ocean carbon inventory"

by

A. J. Watson et al.

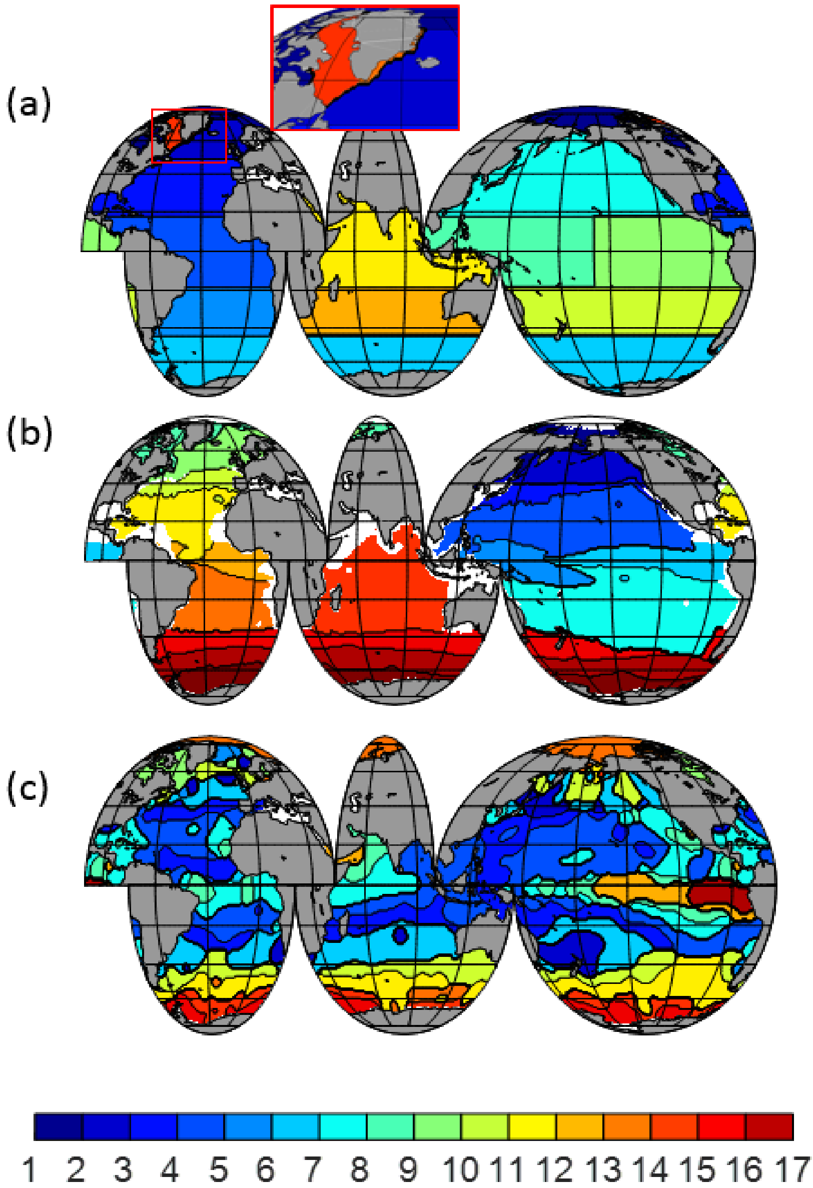

Supplementary Figure 1

Three area divisions of the world ocean used to group observations before interpolation: (a) Latitudinal regions in ocean basins, as used by the "Transcom 3" atmospheric inversion project[1]. In addition to the 12 major regions used there, two small areas east and west of Greenland were added (see inset). These low-temperature waters have substantial fCO_2 data and show as distinct from adjacent waters. (b) The mean positions of 17 biogeochemical zones as derived by Fay and Mckinley[2]. (c) Distribution of 16 regions derived from the SOM technique of Landschützer et al[3] (A monthly climatology of distributions averaged over 1992-2018 was derived in this method: the distribution shown is for October.)

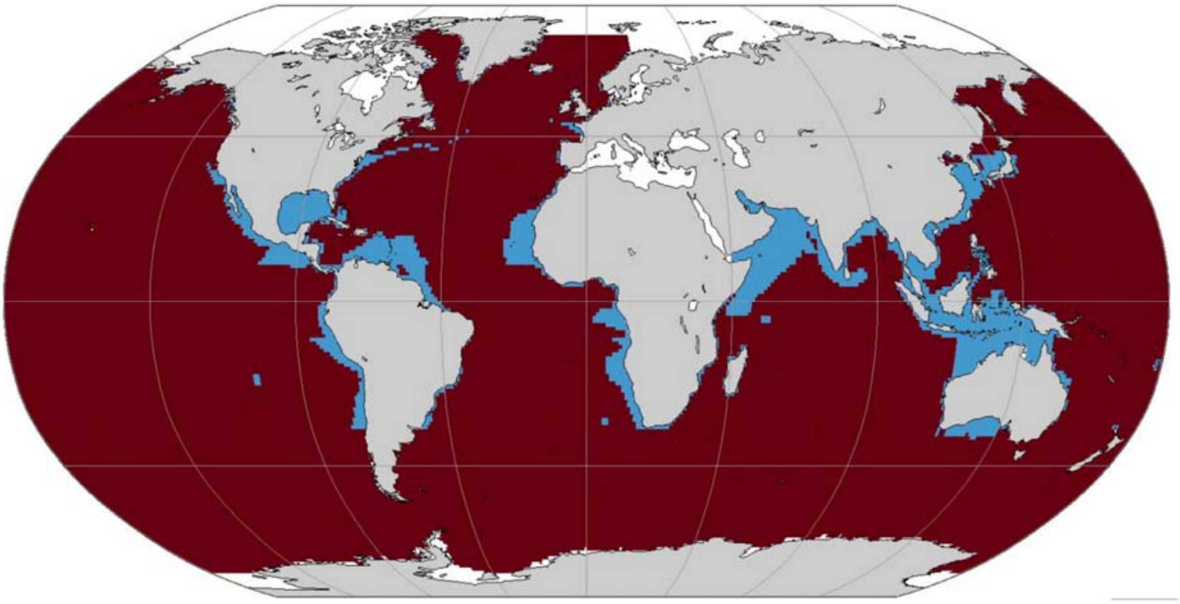

Supplementary Figure 2

Ocean regions included in calculations: areas included in figure 2 main text, and used to calculate uncertainties, are shown in dark red. Ocean regions not classified by Fay and Mckinley'scheme of biomes[2] but included in the other area division methods are shown in blue. These are included in the calculations of figure 3 and Table 2 in the text and Supplementary Table 2 below, which use the FFN-SOM method. White areas are excluded in all calculations.

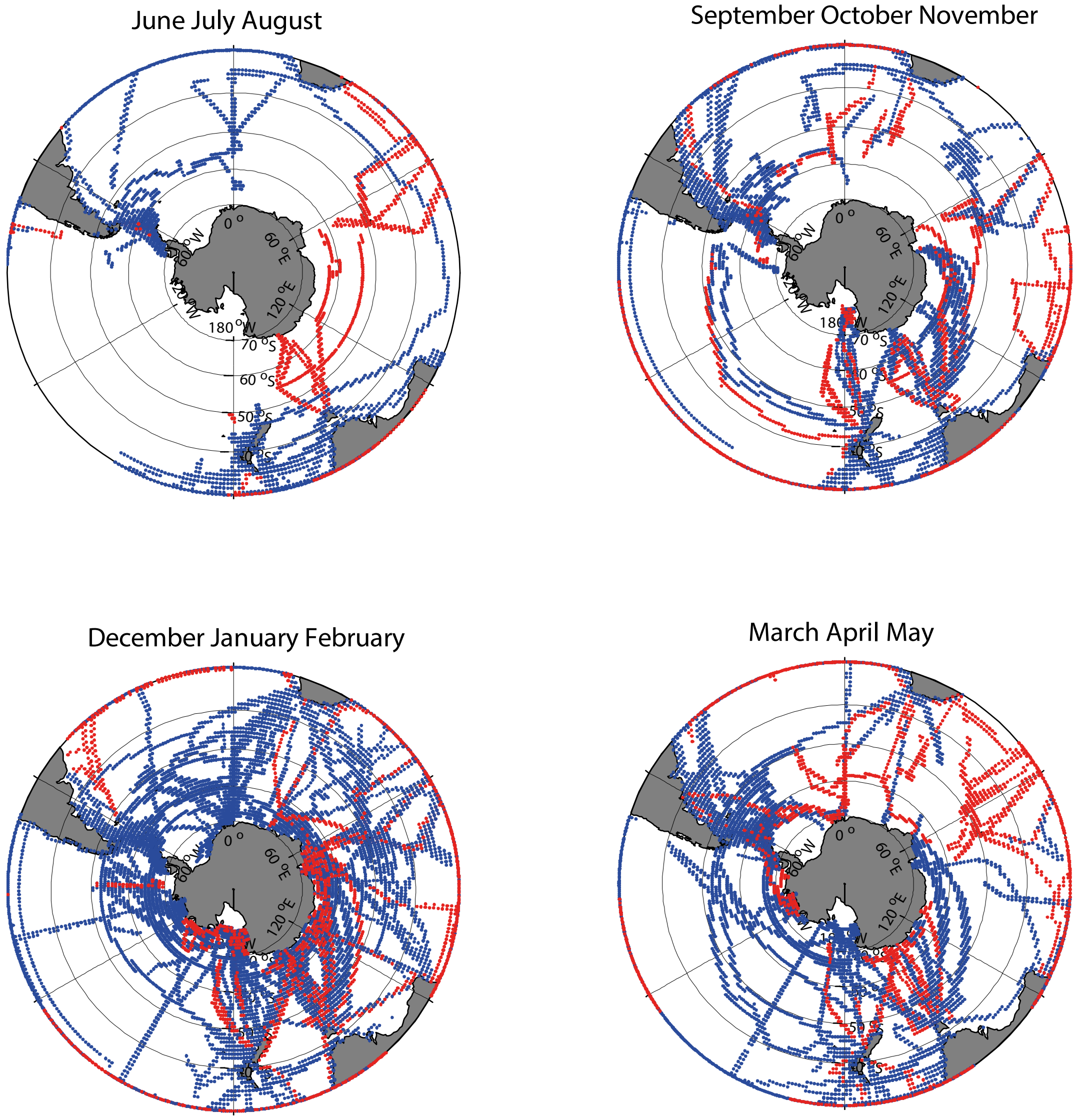

Supplementary Figure 3:

Location of data south of latitude 30 degrees S in the gridded SOCATv2019 product, divided seasonally and pre-and post- 2000. Data from the years 1992-2000 in red. Data from years 2001-2016 in blue. Data coverage is sparse in winter (June-August), with almost none available in the Pacific in either time period. In the Atlantic few winter data are available pre-2000 except around Drake Passage, and in the Indian Ocean few data are available after 2000.

Supplementary Table 1

Sources of data used in the interpolation schemes.

Variable	Data source	Reference	Resolution
Sea surface fCO2.	https://www.socat.info/index.php/data-access/	SOCAT V2019: Pfeil et al [4,5]	Individual data, and monthly 1x 1 degree grid
Sea surface temperature (for interpolation schemes)	https://www.metoffice.gov.uk/hadobs/hadisst/	https://www.metoffice.gov.uk/hadobs/index.html	monthly, 1 x 1 degree grid
Sea surface temperature (for correction to subskin temperature, and gas exchange calculations)	https://www.esrl.noaa.gov/psd/data/gridded/data.noaa.oisst.v2.html	NOAA/OAR/ESRL PSD, Optimum Interpolation SST V2<br>Banzon et al, [6]	monthly, 1 x 1 degree grid
Ice cover	https://www.metoffice.gov.uk/hadobs/hadisst/	https://www.metoffice.gov.uk/hadobs/hadisst/	monthly, 1 x 1 degree grid
Sea surface salinity	http://apdrc.soest.hawaii.edu/erddap/index.html	ECCO2 reanalysis, Wunsch et al,[7]	daily, 0.25 x 0.25 degrees
Mixed layer depth	http://apdrc.soest.hawaii.edu/erddap/index.html	ECCO2 reanalysis, Wunsch et al[7]	3-daily, 0.25 x 0.25 degrees
Marine boundary layer atmospheric XCO2	ftp://aftp.cmdl.noaa.gov/data/trace_gases/co2/flask/surface/.	NOAA ESRL:<br>Duglokencky et al[8]	zonal average, approx weekly x 41 latitudes
sea level atmospheric pressure	www.esrl.noaa.gov/psd/data/gridded/data.ncep.reanalysis.surface.html	NCEP reanalysis:<br>Kalnay et al[9]	monthly, 2.5 x 2.5 degrees
Winds	www.remss.com/ccmp	CCMP product:<br>Atlas et al[10]	6-hourly, 0.25 x 0.25 degrees

Supplementary Table 2

Comparison of annual global CO_2 uptake by the ocean in PgC yr^{-1}, for this work and for earlier estimates (negative values indicate net flux into ocean). Uncertainties when quoted are 2-σ, or twice interquartile range for Gruber et al, ref 13. Estimates by Landschützer et al and Rödenbeck et al are updated periodically: these values are those from the data file linked to Le Quéré et al [11].

	Landschützer et al [3]	Rödenbeck et al [12]	This work	Gruber et al[13]	Global Carbon Project [11]
	**ocean-atmosphere surface flux**				
**1992-2000**	-1.1	-1.7	-2.0±0.4	-	-
**2001-2010**	-1.4	-1.8	-2.2±0.3	-	-
**2011-2017**	-2.0	-2.3	-3.0±0.5	-	-
**1994-2007**	-1.2	-1.6	-2.0 ±0.4	-	-
	**anthropogenic uptake**				
**1992-2000**	-1.7*	-2.1**	-2.6±0.4*	-	-2.0±0.6
**2001-2010**	-2.0*	-2.2**	-2.8±0.3*	-	-2.2±0.6
**2011-2017**	-2.5*	-2.8**	-3.6±0.5*	-	-2.5±0.7
**1994-2007**	-1.8*	-2.1**	-2.5±0.4*	-2.6±0.4	-2.0±0.6

Notes:
*Calculated as surface flux with increase of -0.45 PgC yr-1 to account for pre-industrial flux, and -0.12 PgC yr-1 for Arctic.

** Calculated as surface flux increased by -0.45 PgC yr-1 to account for pre-industrial flux.

Supplementary References

1 Gurney, K. R. *et al.* Towards robust regional estimates of CO2 sources and sinks using atmospheric transport models. *Nature* **415**, 626-630, doi:10.1038/415626a (2002).

2 Fay, A. R. & McKinley, G. A. Global open-ocean biomes: Mean and temporal variability. *Earth Syst. Sci. Data* **6**, 273-284, doi:10.5194/essd-6-273-2014 (2014).

3 Landschützer, P., Gruber, N., Bakker, D. C. E. & Schuster, U. Recent variability of the global ocean carbon sink. *Global Biogeochem. Cy.* **28**, 927-949, doi:10.1002/2014gb004853 (2014).

4 Pfeil, B. *et al.* A uniform, quality controlled Surface Ocean CO_2 Atlas (SOCAT). *Earth Syst. Sci. Data* **5**, 125-143, doi:10.5194/essd-5-125-2013 (2013).

5 Sabine, C. L. *et al.* Surface Ocean CO2 Atlas (SOCAT) gridded data products. *Earth Syst. Sci. Data* **5**, 145-153, doi:10.5194/essd-5-145-2013 (2013).

6 Banzon, V., Smith, T. M., Chin, T. M., Liu, C. & Hankins, W., 2016. A long-term record of blended satellite and in situ sea-surface temperature for climate monitoring, modeling and environmental studies. . *Earth Syst. Sci. Data* **8**, 165–176, doi:10.5194/essd-8-165-2016 (2016).

7 Wunsch, C., Heimbach, P., Ponte, R. M. & Fukumori, I. The global general circulation of the ocean estimated by the ECCO-Consortium. *Oceanography* **22**, 88-103, doi:10.5670/oceanog.2009.41 (2009).
8 Dlugokencky, E. J., Thoning, K. W., Lang, P. M. & P.P., T. NOAA Greenhouse Gas Reference from Atmospheric Carbon Dioxide Dry Air Mole Fractions from the NOAA ESRL Carbon Cycle Cooperative Global Air Sampling Network. *Data Path: ftp://aftp.cmdl.noaa.gov/data/trace_gases/co2/flask/surface/*. (2017).
9 Kalnay, E. *et al.* The NCEP/NCAR Reanalysis Project. . *Bull. Amer. Met. Soc.,* **77**, 437 - 471 (1996).
10 Atlas, R. *et al.* A cross-calibrated, multiplatform ocean surface wind velocity product for meteorological and oceanographic applcations. *Bull. Amer. Meteor. Soc.* **92**, 157-174 (2011).
11 Le Quéré, C. *et al.* Global Carbon Budget 2017. *Earth Syst. Sci. Data* **10**, 405-448, doi:10.5194/essd-10-405-2018 (2018).
12 Rodenbeck, C. *et al.* Global surface-ocean pCO_2 and sea-air CO_2 flux variability from an observation-driven ocean mixed-layer scheme. *Ocean Sci.* **9**, 193-216 (2013).
13 Gruber, N. *et al.* The oceanic sink for anthropogenic CO_2 from 1994 to 2007. *Science* **363**, 1193-1199, doi:10.1126/science.aau5153 (2019).
