# Supplementary material for: Revised estimates of ocean-atmosphere CO2 flux are consistent with ocean carbon inventory
Source: Nat Commun. 2020 Sep 4;11:4422. doi: 10.1038/s41467-020-18203-3 (PMC7474059; doi:10.1038/s41467-020-18203-3)
Supplement: Supplementary file 2 — Peer Review File [file 41467_2020_18203_MOESM2_ESM.docx]

Peer Review Information

**Journal:** Nature Communications

**Manuscript title:** Click here to enter text.

**Corresponding author name(s):** Click here to enter text.

**Editorial notes:**

**Transferred manuscripts**

This manuscript has been previously reviewed at another journal that is not operating a transparent peer review scheme. This document only contains reviewer comments and rebuttal letters for versions considered at *Nature Communications*.

**Reviewer comments & decisions:**

**Reviewer comments, first version:**

Reviewer #1 (Remarks to the Author):

Review of “Larger estimates of ocean‐atmosphere CO2 flux are consistent with ocean carbon inventory”, by A.J. Watson, U. Schuster, J.D. Shutler, T. Holding, I.G.C. Ashton, P. Landschützer, D.K. Woolf and L. Goddijn‐Murphy

Significance: The global and regional estimates of the air/sea carbon flux are critical to understanding the global carbon cycle and its response to the anthropogenic trend. The authors have done an incredible job laying out the sources of uncertainty and/or error in previous assessments. Their new assessment removes several of the outstanding differences between pre- and post-anthropogenic quantifications of the carbon cycle.

Summary: This study uses the SOCAT pCO2 surface ocean database to re-assess the global and regional air/sea CO2 fluxes explicitly accounting for some known biases that were typically ignored in previous assessments. Specifically, the authors correct for temperature differences between the measured pCO2 (usually a few meters below the surface) and the pCO2 in the molecular skin layer (the top 100 microns), as well as the thermal skin layer (the top 1000 microns), including the observed salinity difference as well. The authors use three different interpolation methods to account for missing data in space and time, and while there are still large areas with virtually no data (e.g. the South Pacific during the austral winter), the three methods generally agree. The authors conclude that the yearly rate of uptake of carbon by the ocean is as much as double the previous estimates, and the global uptake between 1992-2018 is about 50% larger. The authors also indicate that their new quantification agrees much better with independent estimates of ocean carbon inventories.

Assessment: This a careful and thorough study. They attribute uncertainties and errors in previous assessments to differences in the temperature at which the air/sea carbon gradient exists and to differences in the calculated gas exchange rates. Their comparison of three different “gap-filling” procedures is the best that can be done until a global biogeochemical float array similar to Argo can be implemented.

One small quibble I have is that they attribute the temperature differences in the thermal skin to outgoing longwave and make no mention of the wind-driven latent heat loss at the surface.

This paper is sure to become a classic, oft-cited study.

Recommendation: Accept and publish.

Major Points:

Minor Points:
1) Line 18: This isn’t a complete sentence. What does this work have “in common with recent studies”? S/b something like: “In common with recent studies evaluating air/sea carbon fluxes, …”
2) Line 24: The word “this” is ambiguous – I assume it means the “concentration at the base of [molecular diffusion] layer”?
3) Figure 2: The shading indicating the uncertainty is nearly impossible to see.


Reviewer #2 (Remarks to the Author):

Review of ‘Larger estimates of ocean-atmosphere CO2 flux are consistent with ocean carbon inventory’ by Watson et al.

This is a potentially significant paper which finds that fully accounting for temperature gradients within the surface layer of the ocean results in a substantially higher estimate of atmosphere-ocean CO2 flux than previous estimates, which is more consistent with global estimates of change in ocean carbon inventory. I think that this paper may be suitable for publication after revision.

Main comments
My main concern with the paper is that the methods and implications of the study are not sufficiently clearly explained. Nature Communications is an interdisciplinary journal with a readership across the sciences. As a climate scientist who is not a specialist in the area of ocean carbon fluxes I found the manuscript hard to understand, and basic concepts not clearly explained. I think the manuscript would benefit from being made more accessible at least to a broader climate science audience.

Specific comments:
Introduction and abstract: These parts of the manuscript should give some motivation for why accurate measures of the atmosphere-ocean CO2 flux are important, and give the basic approach to how these fluxes are calculated. The meaning of ‘fugacity’ should be described when first used. The abstract should lead off with background, rather than starting immediately with a description of the dataset used.
Ln 27: The OCEANFLUX project is mentioned without any description. If the project is critical to the manuscript, give more details and describe. If not, then the project doesn’t need to be named, and a reference to the papers describing the results should be enough.
Ln 43: Specify the variable which is being considered here.
Ln 87-88: How is ‘geostatistical analysis’ different to what is being done here? Is the difference that the geostatistical analysis attempts to model the uncertainties explicitly?
Ln 106-107: Does excluding some regions affect the main results? Can you give a better justification for excluding some regions beyond just that it makes it easier to compare between methods?
Table 2: The label ‘basin’ is unclear, especially as applied to the ‘global’ column. ‘Total’ would be better.
Ln 363-365: Where do these differences of 0.17K and 0.1 psu come from? This isn’t clearly explained in the text.
Figure 2: The solid blue line is not described in the caption.
Ln 233: What is ‘XCO2’?


Reviewer #3 (Remarks to the Author):

In “Larger estimates of ocean-atmosphere CO2 flux are consistent with ocean carbon inventory,” Watson et al. apply temperature and salinity adjustments to ship-board fCO2 measurements to account for differences between “inlet”, subskin, and thermal skin temperatures and salinities. These impacts have been previously documented in papers discussing the air-sea flux, but this is the first time they have been used in some of the same global air-sea flux calculations that underpin our understanding of the ocean’s role in the carbon cycle. Overall this is an important issue that needs to be considered in CO2 flux calculations and one that could have a significant impact on our picture of CO¬2 uptake by the ocean.

My main comments concern how this work fits into other work looking at the global carbon cycle and whether this requires a larger-scale re-thinking of the partitioning of carbon between the ocean and land or if this fits within earlier estimates. The authors partially address this in the section titled “New surface flux of CO2 comparison with interior observations, but I think a few small additions/clarifications would greatly help the reader take away the correct message.

The main takeaway is that consideration of temperature and salinity differences between shipboard sample intakes and the ocean skin increases CO2 uptake estimates derived from surface measurements by ~0.8 Pg C yr-1, a substantial change, especially in recent years when the difference appears to be over 1 Pg C yr-1. This would increase the cumulative ocean uptake from 43 to 67 Pg C from 1992-2018. The authors then state that this updated flux better matches the Gruber et al. 2019 interior anthropogenic accumulation estimates, when considering the pre-industrial, natural ocean source.

What is confusing is that the Gruber et al. 2019 paper explicitly says that their interior change estimates (~31 PgC from 2004-2007) are in agreement with the anthropogenic accumulation derived from Global Carbon Project (Le Quéré et al. 2018, ~26.8 Pg C over the 1994-2007 period). The Le Quéré et al. 2018 values are derived from ocean models that are in good agreement with the Landschützer and Rödenbeck products during the 1990s, i.e. roughly the same period as the ocean interior comparison study. And yet these are the two products that the current study uses as an example of interior/surface flux mismatch. What would be helpful would be another table that compares the various products by decade and for the 1994-2007 period, looking at surface flux and interior accumulation for the current study, the Landschützer/Rödenbeck products, and the Global Carbon Budget values. These should explicitly state whether they represent anthropogenic or contemporary flux estimates and could be in the extended data section, but a few more sentences of discussion could go a long way toward clarifying whether this represents a whole-scale re-writing of the ocean carbon budget or if this indicates that the ocean only models are working well while the surface flux estimates have been a bit biased. This would also provide explicit flux values from this paper for future comparison, which I think are currently missing. I think clarifying the areas of agreement/disagreement with prior work is important for this paper as it initially seems to challenge the broad picture of the ocean’s role in the carbon budget but on closer inspection it appears more complicated.


Other comments:

Title – doesn’t indicate what you actually did

63- Concentration difference uncertainty dominated by interpolation – is that before or after you apply the corrections in this study?

75 – “three different schemes” – perhaps specify “spatial clustering schemes” as it isn’t clear until later how you are dividing the global data.

98-100 - You note that the neural network has much greater flexibility and therefor is better at fitting the training dataset. Was any data withheld? Otherwise it seems you do not include all of the mapping uncertainty, just the ability to fit available data, which should be noted, perhaps in the table.

105 – hard to agree with the Southern Hemisphere results being “convergent” after 2000. Maybe for the period ~2003-2010, but by 2010 there is a ~1 PgC yr-1 spread that continues to increase.

Figure 2 – For each method there are three dashed lines, which represent the different spatial clustering schemes, but there is no way to distinguish the lines from each other. Is there some way to fix this or perhaps note which schemes yield higher/lower flux estimates (assuming this is consistent between approaches).

Figure S3 is too low resolution.

**Author rebuttal, first version:**

Reviewer #1 (Remarks to the Author):

Significance: The global and regional estimates of the air/sea carbon flux are critical to understanding the global carbon cycle and its response to the anthropogenic trend. The authors have done an incredible job laying out the sources of uncertainty and/or error in previous assessments. Their new assessment removes several of the outstanding differences between pre- and post-anthropogenic quantifications of the carbon cycle.

Summary: This study uses the SOCAT pCO2 surface ocean database to re-assess the global and regional air/sea CO2 fluxes explicitly accounting for some known biases that were typically ignored in previous assessments. Specifically, the authors correct for temperature differences between the measured pCO2 (usually a few meters below the surface) and the pCO2 in the molecular skin layer (the top 100 microns), as well as the thermal skin layer (the top 1000 microns), including the observed salinity difference as well. The authors use three different interpolation methods to account for missing data in space and time, and while there are still large areas with virtually no data (e.g. the South Pacific during the austral winter), the three methods generally agree. The authors conclude that the yearly rate of uptake of carbon by the ocean is as much as double the previous estimates, and the global uptake between 1992-2018 is about 50% larger. The authors also indicate that their new

quantification agrees much better with independent estimates of ocean carbon inventories.

Assessment: This a careful and thorough study. They attribute uncertainties and errors in previous assessments to differences in the temperature at which the air/sea carbon gradient exists and to differences in the calculated gas exchange rates. Their comparison of three different “gap-filling” procedures is the best that can be done until a global biogeochemical float array similar to Argo can be implemented.

One small quibble I have is that they attribute the temperature differences in the thermal skin to outgoing longwave and make no mention of the wind-driven latent heat loss at the surface.

We’ve added a reference to the latent heat flux where we first discuss the cool skin, (revised text Line 34-35).

This paper is sure to become a classic, oft-cited study.
Thanks very much for your supportive review!

Recommendation: Accept and publish.
Major Points:

Minor Points:

1) Line 18: This isn’t a complete sentence. What does this work have “in common with recent studies”? S/b something like: “In common with recent studies evaluating air/sea carbon fluxes, ...”

Done (Line 20 in the revision).

2) Line 24: The word “this” is ambiguous – I assume it means the “concentration at the base of [molecular diffusion] layer”?

We have added the word “concentration” here to clarify the meaning (Line 27 in revision)

3) Figure 2: The shading indicating the uncertainty is nearly impossible to see. Thanks for pointing this out. We have redrawn the figures, and darkened the shaded areas to make these clearer.

Reviewer #2 (Remarks to the Author):

Review of ‘Larger estimates of ocean-atmosphere CO2 flux are consistent with ocean carbon inventory’ by Watson et al.

This is a potentially significant paper which finds that fully accounting for temperature gradients within the surface layer of the ocean results in a substantially higher estimate of atmosphere-ocean CO2 flux than previous estimates, which is more consistent with global estimates of change in ocean carbon inventory. I think that this paper may be suitable for publication after revision.

Main comments

My main concern with the paper is that the methods and implications of the study are not sufficiently clearly explained. Nature Communications is an interdisciplinary journal with a readership across the sciences. As a climate scientist who is not a specialist in the area of ocean carbon fluxes I found the manuscript hard to understand, and basic concepts not clearly explained. I think the manuscript would benefit from being made more accessible at least to a broader climate science audience.

We’ve responded to this criticism by revising both the initial paragraphs and final paragraphs in the main text, as described further below.

Specific comments:

Introduction and abstract: These parts of the manuscript should give some

motivation for why accurate measures of the atmosphere-ocean CO2 flux are important, and give the basic approach to how these fluxes are calculated. The meaning of ‘fugacity’ should be described when first used. The abstract should lead off with background, rather than starting immediately with a description of the dataset used.

We agree this is needed. We have rewritten the first part of the abstract to give the most important motivation and rationale for accurate ocean flux estimates. We have removed the reference to “fugacity” in the abstract, instead rewriting in terms of “CO2 concentrations” which conveys the sense better to non-specialists. When we introduce fugacity (revision, lines 20-21), we describe it as “closely equivalent to partial pressure” which hopefully makes it more easily understood. When we first use the symbol “fCO2”, we now define it (Revised text line 28).

A precise definition of fugacity is quite complex and not very helpful– it would confuse non-specialists if put in the main text. We have however added a sentence in the first paragraph of the methods (revision lines 186-188) that reiterates that fugacity is approximately the same as partial pressure but is technically the correct variable to calculate concentrations and fluxes, and gives a reference for this (Weiss, 1974).

To clarify and expand on the implications of our results we have re-written the two last paragraphs of the main text, (revised text lines 164-179). We say there that our revision of the ocean surface flux, now consistent with the independent interior estimate of Gruber et al, means that the ocean observations now both give a similar estimate of ocean uptake, revealing that models significantly underestimate it. The discrepancy with the Global Carbon Project models gets larger towards the present, approaching 1 PgC yr^-1^ since 2010. On the suggestion of reviewer 3, our revision has included adding an extra table into the extended data (shown in red, page 5 of revised extended data) that compares in more detail the fluxes we calculate with other estimates of the ocean sink, and illustrates this clearly,

Ln 27: The OCEANFLUX project is mentioned without any description. If the project is critical to the manuscript, give more details and describe. If not, then the project doesn’t need to be named, and a reference to the papers describing the results should be enough.

We have removed the in-text reference to OCEANFLUX but retained an acknowledgement to it.

Ln 43: Specify the variable which is being considered here.
We now specify “surface fCO2” (revised text line 46).

Ln 87-88: How is ‘geostatistical analysis’ different to what is being done here? Is the difference that the geostatistical analysis attempts to model the uncertainties explicitly?.

We were referring here to techniques that apply the formal methods described in texts on geostatistics, such as objective analysis and kriging, to interpolation of surface fCO2 data (the Jones et al, and Woolf et al references are examples). We have clarified this in the text (revised text line 91).

The reviewer is right -- in this approach a statistical model for the data is assumed that allows the uncertainty to be calculated explicitly.

Ln 106-107: Does excluding some regions affect the main results? Can you give a better justification for excluding some regions beyond just that it makes it easier to compare between methods?

For figure 2 We exclude a few regions for comparison purposes because one of the clustering techniques uses biomes defined by Fay and Mckinley, in which some regions are not classified. We have redrawn Figure S2 to make it easier to see these regions. The omissions alter global flux by only 0.04 PgC yr-1, which is very small on the scale of figure 2. We have added a sentence to this effect (revised text line 111). The estimates in figure 3 and Table 2 do not exclude these areas. The Arctic is the main region excluded in all calculations, because there is so little data there. .

Table 2: The label ‘basin’ is unclear, especially as applied to the ‘global’ column. ‘Total’ would be better.

Done

Ln 363-365: Where do these differences of 0.17K and 0.1 psu come from? This isn’t clearly explained in the text.

We have now cited references for these figures (revised text line 51 and again at line 404). The temperature comes from the global satellite analysis by Donlon et al, (2002) and the salinity estimate is from Woolf et al, 2016.

Figure 2: The solid blue line is not described in the caption.

Hopefully this is now clear: we have redrawn the figures, in response to this query and the other revewers’ comments. The nine individual coloured lines can now be identified to each of the nine methods and the figure caption has been rewritten to explain all the lines (revised text lines 408- 414).

Ln 233: What is ‘XCO2’?

This should have been defined before first use, as we now do here. It is the

atmospheric mixing ratio of CO2. (revised text line 250).

Reviewer #3 (Remarks to the Author):

In “Larger estimates of ocean-atmosphere CO2 flux are consistent with ocean carbon inventory,” Watson et al. apply temperature and salinity adjustments to ship­board fCO2 measurements to account for differences between “inlet”, subskin, and thermal skin temperatures and salinities. These impacts have been previously documented in papers discussing the air-sea flux, but this is the first time they have been used in some of the same global air-sea flux calculations that underpin our understanding of the ocean’s role in the carbon cycle. Overall this is an important issue that needs to be considered in CO2 flux calculations and one that could have a significant impact on our picture of CO¬2 uptake by the ocean.

My main comments concern how this work fits into other work looking at the global carbon cycle and whether this requires a larger-scale re-thinking of the partitioning of carbon between the ocean and land or if this fits within earlier estimates. The authors partially address this in the section titled “New surface flux of CO2 comparison with interior observations, but I think a few small additions/clarifications would greatly help the reader take away the correct message.

The main takeaway is that consideration of temperature and salinity differences between shipboard sample intakes and the ocean skin increases CO2 uptake estimates derived from surface measurements by -0.8 Pg C yr-1, a substantial change, especially in recent years when the difference appears to be over 1 Pg C yr-1. This would increase the cumulative ocean uptake from 43 to 67 Pg C from 1992­2018. The authors then state that this updated flux better matches the Gruber et al. 2019 interior anthropogenic accumulation estimates, when considering the pre­industrial, natural ocean source.

What is confusing is that the Gruber et al. 2019 paper explicitly says that their interior change estimates (-31 PgC from 2004-2007) are in agreement with the anthropogenic accumulation derived from Global Carbon Project (Le Quéré et al. 2018, -26.8 Pg C over the 1994-2007 period). The Le Quéré et al. 2018 values are derived from ocean models that are in good agreement with the Landschützer and Rödenbeck products during the 1990s, i.e. roughly the same period as the ocean interior comparison study. And yet these are the two products that the current study uses as an example of interior/surface flux mismatch.

We fully agree with the reviewer that there is something rather odd here!

Gruber et al’s estimate for the anthropogenic uptake of the oceans 1994-2007 is 33.7 ±4 PgC (see their Table 2 line 3a, or Table I Global total). The equivalent figure in the budget given by the Global Carbon project (GCP) is 26.1 PgC (we have used the Excel table linked to the paper by Le Quéré et al, 2018). Gruber et al give an uncertainty of 4 PgC which is twice the interquartile range of the cases they

consider, comparable therefore to our 2-σ (-95% confidence) interval. The GCP papers give uncertainties but they are 1-σ. The GCP ocean sink is the mean of seven models that range from 21 to 33 PgC for 1994-2007, with a standard deviation of 3.8 PgC, so the 2-σ uncertainty would be twice this, ±7.6 PgC. The Gruber et al and GCP estimates are, therefore, only consistent because the GCP model-based value has such a wide uncertainty, lying between 19 and 33 PgC with something like 95% confidence. Gruber et al say their study “confirms” the GCP estimate. However, the GCP central estimate lies well outside the Gruber uncertainty range. They are consistent only in that Gruber et al’s range of values has some overlap with the upper tail of the GCP estimate at 2-σ uncertainty.

By contrast, the central GCP value of 26 PgC averaged over this period is similar to the Landchützer and Rödenbeck surface-observation-based estimates, once these are adjusted for the pre-industrial natural carbon efflux from the oceans to be comparable with the anthropogenic estimates.

Our revision of the surface observation fluxes is considerably higher than those earlier studies, and is very consistent with the Gruber et al value. Like Gruber et al, our work also strongly suggests that the ocean model estimate used by the GCP is too low, by around 0.5 PgC yr^-1^ in 2000 (and by more than that recently). A major conclusion of our paper is that the observational studies based on two independent data sets now closely agree with one another, and both have smaller uncertainties than the model estimates used by the GCP.

What would be helpful would be another table that compares the various products by decade and for the 1994-2007 period, looking at surface flux and interior accumulation for the current study, the Landschützer/Rödenbeck products, and the Global Carbon Budget values. These should explicitly state whether they represent anthropogenic or contemporary flux estimates and could be in the extended data section,

but a few more sentences of discussion could go a long way toward clarifying whether this represents a whole-scale re-writing of the ocean carbon budget or if this indicates that the ocean only models are working well while the surface flux estimates have been a bit biased. This would also provide explicit flux values from this paper for future comparison, which I think are currently missing.

We have followed all these suggestions, which we agree substantially add to the value of the paper.

1) We have added the suggested table into the extended data (page 5 of revised file) providing fluxes averaged over decadal periods for comparison. It is also our intention to make a data product available similar to the Landschützer and Rödenbeck products, as a resource for researchers.
2) We have added a discussion on this into the final section (revised text lines 164-179). An implication of our work is that most carbon models underestimate the ocean uptake and this requires revision of ocean and probably also land fluxes, and we recommend ocean modellers should address the issue. The adjustment is ~ 0.5-0.6 PgC yr^-1^ to the GCP preferred estimate at around the year 2000, and grows larger towards the present day. This is a substantial change, something more than a minor bias.

I think clarifying the areas of agreement/disagreement with prior work is important for this paper as it initially seems to challenge the broad picture of the ocean’s role in the carbon budget but on closer inspection it appears more complicated.

Agreed, and thank you for the helpful suggestions.

(We have updated figure 3 to use numbers from Le Quéré et al (2018) rather than Le Quéré et al (2016), consistent with the new table – note the Rödenbeck values have changed somewhat between the two as a result.)

Other comments:

Title – doesn’t indicate what you actually did

We have substituted “Revised” for “Larger” in the title, which remains within the

journal guidelines on title but is clearer on what we actually did.

63- Concentration difference uncertainty dominated by interpolation – is that before or after you apply the corrections in this study?

This remains true of the data set after the corrections for systematic error applied here.

75 – “three different schemes” – perhaps specify “spatial clustering schemes” as it

isn’t clear until later how you are dividing the global data.

Done (revised text line 77),

98-100 - You note that the neural network has much greater flexibility and therefor is better at fitting the training dataset. Was any data withheld? Otherwise it seems you do not include all of the mapping uncertainty, just the ability to fit available data, which should be noted, perhaps in the table.

The method used is described by Landschützer et al (2014), and in more detail in Landschützer et al (2013), which we now cite in the revised paper when we first introduce the FFN method (revised text line 83).

To answer the question in brief: yes, data were withheld. Overfitting – in which the network fits not only predictable trends but also the noise in the data – was controlled by establishing the minimum useful size of the network during a pre-training phase. The data in each cluster were then repeatedly randomly divided into training and validation sets, followed by training a network until it passed validation. The final output was the average of 10 such networks. It is true that all of the data were at some point involved in the training since they were repeatedly randomly divided. In the above publications, the method was also tested with completely independent data from time-series stations, observations not included in SOCAT and observations withheld for 5-year time periods (see e.g. Landschützer et al 2014), and found to perform well.

105 – hard to agree with the Southern Hemisphere results being “convergent” after 2000. Maybe for the period ~2003-2010, but by 2010 there is a ~1 PgC yr-1 spread that continues to increase.

“Convergent” was perhaps a too strong a word. We have reworded this to “ ...and there is good agreement also for the southern hemisphere for much of the period after 2000”. (revised text lines 108-109)

Figure 2 – For each method there are three dashed lines, which represent the different spatial clustering schemes, but there is no way to distinguish the lines from each other. Is there some way to fix this or perhaps note which schemes yield higher/lower flux estimates (assuming this is consistent between approaches). To address this we have redrawn the figure using different line styles for the three types of spatial clustering, so it is now possible to distinguish each of the nine methods by their combination of colour and line style. (Revised text lines 409-414).

Figure S3 is too low resolution.
We have corrected this.

**Reviewer comments, second version:**

Reviewer #1 (Remarks to the Author):

I thank the authors for addressing my minor concerns and their clarifications. I also appreciate their responses to the other reviewers - the final result is even more convincing.

I recommend that this paper be accepted and published as is.


Reviewer #2 (Remarks to the Author):

I am happy that the issues raised in my original review have been fully addressed by the authors, and I now recommend that the manuscript be published.


Reviewer #3 (Remarks to the Author):

I appreciate the authors' willingness to address my earlier comments. The more detailed comparison with prior work will make it much easier for this work to be used by future researchers. This revised manuscript should be accepted and will undoubtedly have a significant impact within the community.

One comment:
Line 178 – I would add “interior” so that this reads: “Due weight should be given to the constraints that interior ocean observations impose in future calculations of global carbon budgets.” I think this more clearly captures your point, as the surface observation-derived fluxes are currently used to assess the ocean models used in the global carbon budget.

**Author rebuttal, second version:**

Reviewer #3 (Remarks to the Author):

I appreciate the authors' willingness to address my earlier comments. The more detailed comparison with prior work will make it much easier for this work to be used by future researchers. This revised manuscript should be accepted and will undoubtedly have a significant impact within the community.

One comment:
Line 178 – I would add “interior” so that this reads: “Due weight should be given to the constraints that interior ocean observations impose in future calculations of global carbon budgets.” I think this more clearly captures your point, as the surface observation-derived fluxes are currently used to assess the ocean models used in the global carbon budget.


Yes, but we particularly want ocean models to be assessed taking into account, our *­revised* fluxes, rather than previous estimates. To highlight the need to take both the interior and new surface fluxes into account, we now write: “Due weight should be given to the constraints that ocean interior and surface observations impose when calculating global carbon budgets, and near-surface temperature deviations need to be taken into account when using surface observations to calculate fluxes.” (lines 186-188 in the new version).

**Reviewer comments, third version:**

**Author rebuttal, third version:**
